# The relation between reading and externalizing behavior: a correlational meta-analysis

**DOI:** 10.1007/s11881-024-00307-w

**Published:** 2024-06-29

**Authors:** Sage E. Pickren, Jessica N. Torelli, Anna H. Miller, Jason C. Chow

**Affiliations:** 1 Department of Special Education, Vanderbilt University, Nashville, TN USA; 2 Department of Communication Sciences and Special Education, University of Georgia, Athens, GA USA

**Keywords:** Elementary, Externalizing behavior, Meta-analysis, Reading

## Abstract

Reading proficiency is important because it has life-long consequences and influences success in other academic areas. Many students with behavior problems are poor readers and many students with learning disabilities have more behavior problems than their typical peers. We conducted a correlational meta-analysis to examine the association between reading and externalizing behavior in students ages 5–12. We identified 33 studies that reported 88 effect sizes. Using a random-effects linear regression model with robust variance estimation, we found a significant, negative correlation (*r*= −0.1698, SE = 0.01, *p* < 0.0001) between reading and externalizing behavior. We tested several moderators related to measurement and sample characteristics. We found that rater type, behavior dimension (e.g., aggression), time between longitudinal measurement points, age of the sample, and percentage male of the sample moderated the relation between reading and behavior. Whether the reading assessment measured comprehension or word reading and socioeconomic status of the sample did not moderate the relation. Understanding the association between reading and externalizing behavior has implications for disability identification and intervention practices for children in elementary school. Future research should examine shared cognitive factors and environmental influences that explain the relation between the constructs.

**Supplementary Information:**

The online version contains supplementary material available at 10.1007/s11881-024-00307-w.

## Introduction

Reading proficiency is important because it has life-long consequences and influences success in other academic areas and future employment (Hart et al., 2009; Rabiner et al., 2016a; Sparks et al., 2014). Recent data from the 2022 National Assessment of Educational Progress reveal that 67% of fourth graders in the United States are not performing at a proficient or advanced level in reading (National Center for Education Statistics [NCES], 2022). Acquiring reading proficiency in early elementary school is particularly important because as children age, reading intervention becomes less effective (Lovett et al., 2017). Similarly, behavior problems are more easily remediated when children are young (Cunningham et al., 2023; Fox et al., 2002). Many students with behavior problems are poor readers (Arnold et al., 2005; Nelson et al., 2004) and many students with learning disabilities have more behavior problems than their peers (Horbach et al., 2020). Understanding the association between reading achievement and behavior can help guide educators to use assessment and identification practices that are efficient and develop intervention strategies that support students in reading and behavior.

### Etiology of externalizing behavior and reading deficits

Externalizing behavior is a category of challenging behaviors that are defiant, aggressive, disruptive, or hyperactive (Achenbach, 1999). The biosocial model of development describes the etiology of externalizing behavior as an interaction between nature and nurture (Raine et al., 1997). This model proposes that the risk for developing externalizing behavior stems from a confluence of biological factors (e.g., genetics or prenatal exposure) and psychosocial risk factors such as potential exposure to trauma during childhood (Liu, 2004). Externalizing behaviors are often measured by using direct observation or indirect methods like checklists and surveys; and there are strengths and weaknesses to both methods (Torelli et al., 2022; Yoder & Symons, 2010). Checklist and survey ratings can be self-report or completed by parents, caregivers, or teachers. Raters may differ in how they score externalizing behaviors because of the context in which they know and interact with the child. In multi-rater longitudinal analyses of the development of externalizing behavior, mother ratings of behavior are higher than those of other caregivers and teachers (Miner et al., 2008). Results from this study suggest that the relation between reading and behavior may differ based on how behavior is measured and by whom.

The etiology of reading disability (RD) has similar theoretical foundations, linked to both genetics (DeFries et al., 1987; Wadsworth et al., 2000) and environmental factors, specifically, inadequate instruction (Solis et al., 2014; Vellutino et al., 2004). The Simple View of Reading, a well-accepted model of reading development, proposes that successful reading is built from competence in word decoding and listening comprehension (Hoover & Gough, 1990). Genetics play a large role in word reading, phonological awareness, decoding, and reading comprehension (Christopher et al., 2013; Erbeli et al., 2018; Harlaar et al., 2010). Indeed, several specific genotypes have been proposed as risk factors for dyslexia, a form of RD linked to deficits in word decoding (for a review see: Pennington et al., 2009). Researchers have found that there are both structural and functional brain differences between children with and without RD (Cutting et al., 2013; Rimrodt et al., 2010), and brain differences prior to reading intervention can predict which students will make progress on reading assessments (Aboud et al., 2018). Parental attitudes and behaviors towards reading may represent an overlap of genetic and environmental factors that affect risk. In a longitudinal sample, Nguyen and colleagues (2022) found that parents’ reading difficulty negatively affected their children’s reading skill. Twin studies (e.g., Erbeli et al., 2018) demonstrate some variance in RD is associated with non-shared environmental influences, such as exposure to print in school. Exposure to print at school can influence the Matthew Effect (Stanovich, 1986), a reading model in which good readers become better readers and poor readers become worse readers. Specifically, positive experiences with books expose young children to strong language models and new vocabulary; these exposures predispose children to enjoy reading and, in turn, they choose to read more, providing further exposure to grow their vocabulary and reading skill (e.g., McNamara et al., 2011). When the opposite is true (i.e., students struggle with reading initially), frustration from failure causes avoidance from reading tasks and prevents future learning (e.g., Echols et al., 1996). Children who are genetically predisposed to RD may be less likely to be exposed to positive experiences with books at home and school, amplifying risk for developing RD.

In research, reading can be measured broadly to reflect foundational skills (e.g., nonsense and real word reading) and reading comprehension, with some assessments testing both domains. It is of interest to determine how externalizing behavior relates to foundational skills such as word reading differentially and more complex skills like reading comprehension.

### Cooccurrence of reading disability and externalizing behavior

The documentation of the cooccurrence between reading disability and externalizing behavior disorders dates to about 50 years ago when Rutter and colleagues (1976) studied children on the Isle of Wight. They found that 9- to 11-year-old children with reading delays were over four times as likely than their typical peers to exhibit antisocial (or externalizing) behavior. This finding of heightened levels of externalizing behavior for children with academic deficits has been replicated numerous times (for review, see Hinshaw, 1992). Willcutt and colleagues (2013) found that children with learning difficulties in math, reading, or both exhibited more externalizing behavior than those without learning difficulties. Willcutt and Pennington (2000) had similar findings and also reported sex differences, such that the association between reading disability and externalizing behavior was stronger for boys, whereas the association between reading disability and internalizing behavior (e.g., depression, anxiety) was stronger for girls. The same study found that boys are more likely to display symptoms of impulsivity and hyperactivity among children with RD, and are more likely to be diagnosed with ADHD (Willcutt & Pennington, 2000). A longitudinal analysis of the development of behavior difficulties in students with and without reading difficulties in kindergarten to 5th grade found that children who were later classified with RD did not differ from their typical peers in behavior issues before kindergarten (Horbach et al., 2020). However, after school entry, students who were later classified as RD had increasing externalizing behavior and significantly more behavior, attention, and social problems than their typically developing peers and were more likely to be diagnosed with attention-deficit hyperactivity disorder (ADHD) throughout elementary school. The change in the relation as students progress through school, suggests that this relation may depend on the age of students.

### Theories of comorbidity

Several models of comorbidity have been proposed to explain the common co-occurrence of RD and externalizing behavior : (1) the unidirectional theory, (2) the bidirectional theory, and (3) the multiple deficit model (Hinshaw, 1992; Kulkarni et al., 2021; McGrath et al., 2009). A brief overview of these theories follows.

Two unidirectional theories exist: RD causes behavioral difficulties due to frustration around academic failures, or behavior difficulties cause RD because the disruption to instruction and missing core academic content (Hinshaw, 1992). Some studies demonstrate that early reading predicts later externalizing behavior (Halonen et al., 2006; Bennet et al., 2003) and other studies found that externalizing behavior is negatively associated with academic performance over time (Kremer et al., 2016). The unidirectional theory suggests that intervening on one of the domains (i.e., behavior or academics) would positively affect the other (Morgan et al., 2008); however, evidence for this is mixed. Some studies suggest that students with behavior problems do not respond to otherwise effective core reading instruction (van Dijk et al., 2023). A recent meta-analysis of reading intervention for students with co-occurring RD and exernalizing behavior showed no evidence for improvement in effects of reading or behavior interventions in the other domain (Roberts et al., 2020). Another meta-analysis found that students with comorbid ADHD and RD need reading intervention to improve their reading, not just behavioral and pharmaceutical interventions (Chan et al., 2023).

The bidirectional theory suggests that these two domains are mutually influential. A longitudinal study used the ECLS-K data to determine if 1st-grade reading difficulties predicted problem behavior in 3rd grade and the reverse (i.e., whether 1st-grade problem behavior predicted 3rd-grade reading) while controlling for initial levels of both reading and behavior performance and other variables (i.e., SES, sex, attention, and race), (Morgan et al., 2008). Reading performance in 3rd grade was predicted by low levels of task-focused behavior in 1st grade, and reading performance in 1st grade was a significant predictor of exernalizing behavior in 3rd grade (Morgan et al., 2008). The authors interpreted this result as preliminary evidence of a bidirectional relation between reading and behavior difficulties. Under this bidirectional model, interventions that target both reading and behavioral difficulties simultaneously should improve outcomes for both domains (Morgan et al., 2008). Despite promising correlational evidence, recent experimental studies have shown evidence against this bidirectional relation. After being randomly assigned to a reading intervention or control, upper elementary school students with reading difficulties and problem behavior had smaller effect sizes on reading outcomes than their peers without problem behavior, suggesting the combined intervention was not more effective than the reading-only tutoring (Roberts et al., 2021). A recent meta-analysis found that combined interventions in both reading and behavior for students with RD and problem were not effective in improving behavior outcomes, and were less effective than reading interventions alone in improving reading outcomes (Roberts et al., 2020). Of note, in this meta-analysis there were only 11 group-design studies examined and the studies with combined behavioral and reading interventions used what are considered Tier 1 behavioral supports such as positive reinforcement, not individualized or intensive behavioral supports (Roberts et al., 2020).

In line with the discussion above, another recent meta-analysis found no evidence for a causal link between externalizing behavior and academic achievement, no evidence for the bidirectional effects of the two domains, and weak but conflicting evidence for the unidirectional effect of early behavior problems to later low academic achievement (Kulkarni et al., 2021). These results together with findings from Roberts et al. (2020), suggest that the multiple deficit model (MDM) may be most appropriate to describe the relation between reading and externalizing behavior. The MDM suggests that the two disorders are not causally related, but rather are probabilistically related to shared factors that precede and increase risk for both externalizing behavior and reading achievement (McGrath et al., 2009). The present study aims to meta-analyze the relation between reading and externalizing behavior specifically in a diverse sample of students (i.e., elementary school students with and without RD or behavior disorders). In light of recent research demonstrating that the apparent relation between academic achievement and externalizing behavior is probably not causal (Kulkari et al., 2020; Roberts et al., 2020), a significant relation in the present study would suggest the presence of comorbidity, and that students may have deficits in shared risk factors that affect both the devlelopment of RD and externalizing behavior.

### Shared risk-factors

As introduced above, the relation between reading and externalizing behavior may be explained by a multiple deficit model centered around factors that are related to both domains, such as executive function, attention, or language deficits (Hinshaw, 1992).

#### Executive functions

In a recent meta-analysis in which students with RD were compared to typical readers, significant deficits in several cognitive processes (i.e., processing speed, working memory, attention, inhibition, and short-term memory) were found between the two groups (Peng et al., 2019). Another recent meta-analysis on the longitudinal relationship between executive function and externalizing behavior showed that lower scores on executive function tasks in childhood (i.e., inhibition, working memory, attention shifting, planning, and verbal fluency) are a robust predictor of broad externalizing behavior later in life as well as specific forms of externalizing behavior disorders such as ADHD, conduct disorder, and oppositional defiant disorder (ODD) (Yang et al., 2022). In a cross-sectional model examining comorbidity, processing speed significantly predicted both reading difficulties and an inattention variable associated with ADHD symptomology (McGrath et al., 2009).

#### Attention and hyperactivity

ADHD is a prevalent, high-incidence disability (Wolraich et al., 2019) that is common in both children with reading disability (August & Garfinkel, 1990; Germanò et al., 2010) and behavior disorder (DuPaul & Stoner, 2014). It is often classified into three presentations: excessive hyperactivity, inattention, or combination (Epstein & Loren, 2013; Willcutt et al., 2012). However, the presentations can be difficult to disentangle, with most children exhibiting characteristics of both inattention and hyperactivity (Sobanski et al., 2008).

Comorbid ADHD is a potential shared factor between externalizing behavior and reading, particularly the characteristics of ADHD, attention and hyperactivity/impulsivity. Attention is a known predictor of language and literacy achievement (Chow & Wehby, 2019; Rabiner et al., 2000; Rabine et al., 2016) and is important for academic success in general (Rabiner et al., 2016a). Inattention has been linked to deficits in reading comprehension (Cain & Bignell, 2014) and poor academic outcomes in students with ADHD (Jaekel et al., 2013). There are genetic markers linking RD and general ADHD, and RD to both inattention and hyperactivity (Hart et al., 2010).

Hyperactivity is often considered a dimension of externalizing behavior, alongside aggression and disruptive disorders (Hinshaw, 1987). Though hyperactivity has evidence of independence (or divergent validity) from aggression and conduct disorders (Hinshaw, 1987), many studies examine hyperactivity in conjunction with other behavior disorders (Barkley et al., 1990; Liu, 2004) or study how hyperactivity is associated with other dimensions of externalizing behavior (Greven et al., 2011; Kuja-Halkola et al., 2015). In an all-female sample, hyperactivity and impulsivity in early childhood were significant predictors of externalizing behavior later in life, over and above the effect of inattention, which did not predict externalizing behavior (Ahmad & Hinshaw, 2017). Hyperactivity has been linked to deficits in listening comprehension, rather than reading comprehension in students at risk for ADHD (Cain & Bignell, 2014).

Willcutt and Pennington (2000) examined how attention and hyperactivity were related to reading disability and behavior. Specifically, the presence of an ADHD diagnosis fully mediated the relation between reading disability and aggression, ODD and conduct disorder (CD). In another study, a sample of students aged eight to 15 with comorbid RD and ADHD were significantly more likely to have symptoms of ODD and CD than their RD peers who did not also have ADHD symptomology (Willcutt et al., 2013). Thorbach and colleagues (2020) also demonstrated that the relation between RD and parent reports of behavior was no longer significant after controlling for ADHD diagnosis status, demonstrating that ADHD may explain the relation between reading difficulties and externalizing behavior. Findings from the above experimental studies suggest that comorbid ADHD may be responsible for elevated externalizing symptomology for students with reading disability and that hyperactivity might be a mechanism that connects RD to externalizing behavior.

#### Language

Language has also been identified as a shared factor between externalizing behavior and reading (Hinshaw, 1992). This connection may exist because early reading acquisition requires strong phonological and linguistic processing (Catts et al., 2002) and oral language is critical for reading comprehension (Cutting et al., 2009). Moreover, verbal mediation is a key factor in self-regulation of behavior, which could explain why lower language skills are related to externalizing behavior (Chow et al., 2018), and why children who receive language intervention show reductions in later problem behavior (Curtis et al., 2019). Children with limited language ability may experience frustration when trying to communicate and their frustration may manifest as aggression. Donolato and colleagues (2022) conducted a meta-analysis that examined the level of internalizing and externalizing behavior for students up to the age of 18 with language and specific learning disorders. They found heightened levels of internalizing and externalizing behavior for students with language and specific learning disorders, compared to typical peers.

#### English language learner status

There is evidence that the occurrence of externalizing behavior is related to English language proficiency status. Students who are limited English proficient have higher ratings of externalizing behaviors than their English-proficient peers cross-sectionally but student proficiency status at school entry did not predict externalizing behavior at the end of third grade (Araújo Dawson & Williams, 2008).

#### Socioeconomic status

Socioeconomic status (SES) is a potential antecedent or explanatory variable for externalizing behavior (Hinshaw, 1992). Aggression and conduct disorder have been linked to SES with higher levels of poverty being associated with externalizing behavior (Szatmari et al., 1989: Henninger & Luze, 2013). Low SES has also been associated with reading disability (Chall & Jacobs, 1983), with the home literacy environment serving as an important factor in reading development (Nguyen et al., 2022). Adverse childhood experiences (ACEs) negatively impact both reading performance and externalizing behavior (Morrow & Villodas, 2018) and have a complex association with SES (Jahanshahi et al., 2022; Steele et al., 2016), which could explain failure in both domains from environment.

### Prior systematic reviews and the current meta-analysis

Recent meta-analyses have been published on the causal relation between academic achievement and externalizing behavior more broadly (Kulkarni et al., 2020), reading and internalizing behavior (Francis et al., 2019), internalizing and externalizing behavior and language and learning disorder (Donolato et al., 2022), and the effects of interventions for students with reading difficulties and externalizing behavior (Roberts et al., 2020). The current review complements these contributions by examining the correlation between externalizing behavior and reading specifically, across a wide range of behaviors and students including those with and without RD and clinical behavior disorders.

Several primary studies have documented the correlation between reading and externalizing behavior, yet prior to this paper, there are no correlational meta-analyses that have been conducted to synthesize this relation across studies. Understanding the correlation between the two constructs in both the overall elementary population and in clinical samples helps describe how the skills are related for a typical population and what characteristics might strengthen or weaken the relation.

The purpose of this correlational meta-analysis was to aggregate both the concurrent and longitudinal association between reading and externalizing behavior for elementary-school-aged children. Additionally, we sought to identify potential factors or moderators that strengthened or weakened the relation between reading and externalizing behavior. We asked the following research questions:

1. What is the concurrent and longitudinal association between externalizing behavior and reading performance in elementary school children (ages 5 to 12)?
2. What measurement choices moderate the association between reading and externalizing behavior? Specifically, do behavior rater, type of reading assessment, dimension/type of behavior (e.g., aggression), and/or time between longitudinal measurement points strengthen or weaken the correlation?
3. What child sample characteristics moderate the correlation between reading and externalizing behavior? Specifically, do age, sex, English language learner status, and/or disability status strengthen or weaken the correlation?

Based on prior work, we hypothesize that the relation between reading and externalizing behavior will be negative, such that more externalizing behavior will correlate with lower outcomes on reading measures. For the moderators, we hypothesize that there will be significant variability among studies, and this variability will be associated with some of the theoretical moderators listed in research questions 2 and 3. Specifically, we predicted that the relation would be greater for boys vs. girls, for older children vs. younger children, for students with disabilities vs. typically developing students, and for English language learners vs. native English speakers.

## Methods

### Inclusion and exclusion criteria

We included both published and grey literature (i.e., dissertations) in the review. To be included, studies had to use group design methodology and include a sample of children between the ages five and 12 (or grades Kindergarten through 6th grade). We allowed studies to include participants with a full range of reading ability and behavior, such that children with learning disabilities, language disorders, behavior disorders, and attention deficit hyperactivity disorder (ADHD) were included. We also included participants who were learning English as a second language. We included intervention studies in the review only if pre-intervention data were available. Studies had to report a bivariate correlation between a direct measure of reading performance (e.g., the Woodcock-Johnson Test of Achievement, Schrank & Wendling, 2018) and an indirect measure of externalizing behavior (e.g., The Child Behavior Checklist; CBCL, Achenbach, 1999). Externalizing behavior included dimensions of aggression, conduct problems, disruptive behavior, acting out, antisocial behavior, or externalizing behavior generally (as labeled by the instrument). Behavior measures could be standardized or unstandardized and reported by parent or teacher or by the child themselves. Reading measures could be standardized or unstandardized and assess word reading, (real word reading or nonsense word reading) comprehension (sentence or passage), or a combination of both constructs.

We excluded studies from the review if they used single-case design methodology because correlations are not commonly reported using single-case research. If a study used a proxy for reading (e.g., vocabulary, spelling), only a measure of pre-reading (e.g., print awareness, phonological awareness) or if the reading assessment was conducted in another language besides English, we excluded the study. This decision was made because English is less orthographically transparent than other languages and therefore affects reading acquisition (Ziegler et al., 2010). We excluded studies if they did not disaggregate internalizing behavior from externalizing behavior and instead reported a total behavior score. Though ADHD is a category of externalizing behavior in the DSM-V, correlations between inattention/hyperactivity and reading were not recorded because meta-analyses on this relation already exist (Daucourt et al., 2020; McDougal et al., 2022). However, as stated earlier, samples including students with ADHD were included specifically for moderator analysis. We excluded studies if participants had intellectual disability, developmental disability (e.g., autism spectrum disorder), epilepsy, or visual or hearing impairments. We excluded autism spectrum disorder because of atypical relations between intellectual ability and behavior with academic performance reported in samples with the disorder (Estes et al., 2011).

### Systematic search

We identified studies using two methods: a multi-database electronic search and an ancestral search of included studies. We ran the electronic search conducted in *ProQuest’s Social Science Premium* (which includes the *ERIC* database, PsychINFO, *ProQuest’s Psychology* database, and *Dissertations and Theses Global)*. The search was conducted on July 19th, 2021 and contained the following search terms: [STRICT] ti, ab, su(externali*ing OR aggression OR (aggressive* NEAR/3 behavio*) OR “behavio* problem*”) AND ti, ab(reading) AND ti, ab(child* OR adolescent* OR student* OR school*) AND stype.exact(“Scholarly Journals” OR “Dissertations & Theses”) AND Noft((“primary school*” OR “elementary school*” “elementary age*” “kindergarten*” OR “first grade*” OR “second grade*” OR “third grade*” OR “fourth grade*” OR “fifth grade*” OR “sixth grade*”) OR ((age* OR old* OR year*) NEAR/3 (5 OR 6 OR 7 OR 8 OR 9 OR 10 OR 11 OR 12 OR five OR six OR seven OR eight OR nine OR ten OR eleven or twelve))). This search returned 1,137 unique studies.

**Fig. 1:**
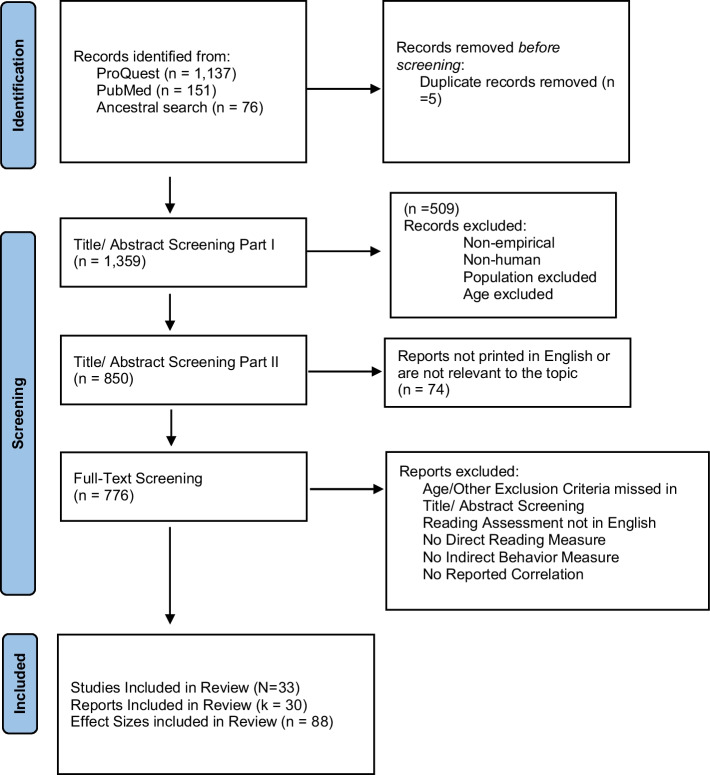
PRISMA diagram

We also ran a search in PubMed on July 19th, 2021 using the following search terms: (“externalizing behavio*“[tiab] OR aggression[tiab] OR aggressiv*[tiab] OR “problem* behavior*“[tiab]) AND (reading[tiab]) AND (“Child“[Mesh] or kindergarten*[tiab] or “age 5”[tiab] or “5 year*”[tiab] or “age 12”[tiab] or “12 year*”[tiab]). This search returned 151 studies with 5 duplicates overlapping with *ProQuest.* See the PRISMA diagram in Fig. 1 for more information about study identification.

### Screening

We screened the resulting 1359 studies first by only viewing the study titles and abstracts with the purpose of removing studies that clearly did not meet the study criteria. We excluded studies if they were (1) non-empirical, such as studies with no data and results in them, qualitative reports, meta-analyses, book reviews, or practitioner papers, (2) non-human, such as studies conducted on animals or cellular research, (3) populations that included participants who met one of the exclusion criteria, such as intellectual disability or blindness, (4) participants were under five years of age or over 12 years of age. The first and second authors, along with two trained undergraduate and graduate research assistants, screened studies. We calculated inter-rater reliability on 20% of randomly selected studies and agreement was 91%. We conducted a second round of title/abstract screening to remove studies that were printed in another language besides English (after no English version was accessible) or that were not relevant to the topic (e.g., studies about only math achievement, child body-mass index, analysis of child drawings) .

Seven hundred seventy-six studies were eligible for full-text screening. We downloaded the full article to ensure studies met the inclusion criteria. The focus of this phase of screening was to check that the study contained appropriate reading and behavior measures and reported a bivariate correlation between the measures. We checked the initial inclusion and exclusion criteria concerning participant population and age and group design methodology. This resulted in the inclusion of 33 studies. See Appendix A for the reference list of included studies.

### Study coding

We coded identifying features of the study and recorded them in an Excel spreadsheet, like the authors name, year, and whether the study was a peer-reviewed published article or a dissertation. We coded studies that included participants from the same national sample (e.g., Early Childhood Longitudinal Studies-Kindergarten; ECLS-K) under the same study identification number for data nesting purposes, which resulted in 30 unique reports. We extracted the number of participants and the bivariate correlation between the direct measure of reading and the indirect measure of behavior from the text or table of the article. Some reports contained multiple correlations, such as an association between word reading and externalizing behavior *and* an association between comprehension and externalizing behavior. In those instances, we extracted all reported correlations and nested them into the single study identification number by using multiple rows in the spreadsheet with the same study number. We coded what instruments were used to measure reading and behavior and classified them according to their constructs. For longitudinal correlations, we reported the direction of the association (i.e., early reading to later behavior *or* early behavior to later reading) and the time lag between the two measurement points. When reported, we also coded demographics of the sample including mean age, percentage that were learning English as a second language, and percentage that was male verses female. For socio-economic status, we coded the percentage that was recruited or characterized as coming from poverty. When reported, we also coded disability characteristics of the sample, including percentage that had diagnoses reading disability, behavior disorder, developmental language delay, and ADHD. See the Supplementary Information for the full coding manual. The first author served as the primary coder and the second author and an undergraduate student served as a second coder. Prior to coding studies for reliability, the second coders were trained on the coding manual and practiced coding three studies (that were not included in IOA) to allow for coaching and feedback. Inter-rater reliability was conducted on 33% of studies (k = 11, effect sizes = 22). Agreement was 89.14% and discrepancies were discussed and rectified.

### Analysis

#### Random-effects linear regression

To conduct the meta-analysis, we used a random-effects linear regression model with robust variance estimation. Robust variance estimation handles study dependencies by clustering standard errors without requiring knowledge of within-study variance (Hedges et al., 2010; Pustejovsky & Tipton, 2022). Our data were also structured hierarchically with effect sizes nested within reports. The random-effects linear model allowed for study identification number to be treated as a random effect and thus accounted for dependencies among effect sizes coming from the same report. We performed the analysis in R (version 4.0.2) using the “metafor” and “robumeta” packages (Fisher & Tipton, 2015; Tipton & Pustejovsky, 2015; Viechtbauer, 2010). We extracted and transformed Pearson *r* correlations to Fisher’s *z* and performed all analyses with the transformed value to normalize and stabilize the sampling variances (Borenstein et al., 2021). Results were then converted back to Pearson’s *r* for interpretation. We meta-analyzed effect sizes from all reports to produce an overall correlation between reading and externalizing behavior. Next, we split the data into concurrent verses longitudinal effect sizes to report an overall correlation for both types of associations. We calculated prediction intervals using the *predict* function in the “metafor” package to estimate prediction intervals for the meta-analyzed effect sizes. Prediction intervals at a 95% confidence interval estimate the range in which a new correlation from an unincluded study is likely to fall within.

#### Moderator analysis

Prior to performing moderator analysis, we calculated several statistics, including Cochran’s Q test for Heterogeneity, to evaluate the heterogeneity between effect sizes in our meta-analysis and therefore justify moderator testing (Cochran, 1950). We also examined I^2^ and tau-squared to determine the proportion of variance that is explainable (not due to error) and the magnitude and distribution of heterogeneity, respectively (Higgins et al., 2003). We did not proceed with moderator analyses for any category that had less than 10 studies, as recommended by Geissbuhler et al. (2021).

We tested four moderators related to measurement choices made in the study (rater of behavior measure, type of reading measure, type of behavior measure, and longitudinal lag). We tested three moderators related to participant characteristics (mean age, percentage male of the sample, and percentage of the sample coming from poverty). For categorical moderators, we reported the *Q*_*m*_-test, which is an omnibus test using a chi-squared distribution. For categorical moderators that were significant, we adjusted the intercept to zero to interpret the correlation estimate for each category. For continuous moderators, we reported the *Beta* estimate and created a plot of the moderator to examine the direction of the relation.

## Results

### Narrative results

#### Description of studies and effect sizes

The 33 studies that were included in the review reported 88 effect sizes (i.e., Pearson correlations). Studies reported between one and eight effect sizes. Several studies used national samples, predominantly the ECLS-K, which resulted in 30 “reports” included in the review or 30 unique identification numbers used for nesting in the analysis. Our sample included 22 peer-reviewed studies and 11 dissertations. Publication dates ranged from 1987 to 2022. We included both concurrent (k = 63) and longitudinal (24) with the majority (18) of longitudinal correlations reporting early behavior to later reading. See Table 1 for further characteristics of studies and their samples, including mean age, sex, percentage of the sample containing special education labels and other demographic information.

**Table 1:** Characteristics of Studies and Participants

			Sample Characteristics							
Study	Published	Effect Size Type	Mean Age/Grade	% Male	% Poverty	% ELL	% DLD	% RD	% BD	% ADHD
Adams, 1999	Peer-Reviewed	Concurrent	3rd Grade	49.7	28.5	0			8.7	
Bierman, 2013	Peer-Reviewed	Concurrent	Kindergarten	.	35					
Bodovski, 2011	Peer-Reviewed	Both	1st Grade	50						
Boetsch, 1996	Dissertation	Longitudinal	1st Grade	53, 50				0, 100		17, 5.5
Boyes, 2018	Peer-Reviewed	Concurrent	3rd Grade	40		0				
Braciszewski, 2007	Dissertation	Concurrent	3rd Grade		73					
Clarke, 2008	Dissertation	Concurrent	Kindergarten	50	28					
El Nokali, 2012	Dissertation	Concurrent	4th Grade	48, 50	29.1					
Feshbach, 1987	Peer-Reviewed	Concurrent	2nd-4th Grade	0, 50, 100						
Finn, 1995	Peer-Reviewed	Longitudinal	4th Grade	51.6						
Fletcher, 2001	Peer-Reviewed	Concurrent	1st grade	.						
Garwood, 2017	Peer-Reviewed	Concurrent	3rd Grade	.	56.2		0.02	0.03	100	
Goldberg, 2004	Dissertation	Concurrent	5th Grade	100						
Goldwater, 2001	Dissertation	Concurrent	3rd Grade	32	27	16				
Gray, 2010	Dissertation	Longitudinal	Kindergarten	.	.		0.5		0.97	
Horan, 2016	Peer-Reviewed	Concurrent	2nd grade	49	61.8					
Jeffrey, 2020	Dissertation	Both	2nd grade	48	84					
Kwon, 2012	Peer-Reviewed	Concurrent	K-3rd Grade	74.8	49	4	11.5		11.1	15.9
Magnuson, 2016	Peer-Reviewed	Concurrent	K-6th Grade							
Mano, 2017	Peer-Reviewed	Concurrent	2nd-5th Grade	0, 100	69			15, 26		100
Martoccio, 2014	Dissertation	Concurrent	4th Grade	48.8	100					
Mesite, 2019	Dissertation	Both	Kindergarten	51		15.82				
Miller, 2006	Peer-Reviewed	Concurrent	3rd Grade	86		0			4.3	93.2
Morgan, 2019	Peer-Reviewed	Both	K-2nd Grade		21	16.2				
Morrison, 1989	Peer-Reviewed	Concurrent	Kindergarten	53.2	34					
NICHD, 2005	Peer-Reviewed	Concurrent	1st Grade		21					
Razza, 2015	Peer-Reviewed	Both	K-3rd Grade	47.8	65					
Sasser, 2015	Peer-Reviewed	Concurrent	3rd Grade		68					
Stormont, 2019	Peer-Reviewed	Both	K-1st Grade		36					
Swanson, 2016	Peer-Reviewed	Both	K-1st Grade	60						
Tang, 2021	Peer-Reviewed	Both	K-1st Grade	51						
Tomblin, 2000	Peer-Reviewed	Concurrent	1st Grade	56.8			35.8			
Wang, 2009	Dissertation	Concurrent	2nd grade	51	9					

*Note. *Both study reports both longitudinal and concurrent effect sizes, *ELL* english language learners, *DLD* developmental language disorder, *RD* reading disability, *BD* behavior disorder, *ADHD* attention deficit hyperactivity disorder. Some studies contained multiple effect sizes and therefore have two values listed for percent male

Most reading measures used in the studies (43 of the 88 effect sizes) were a combination of word reading and comprehension that were often reported as a composite score from multiple subtests (e.g., Woodcock-Johnson Broad Reading comprised of Letter-Word Identification, Reading Fluency, and Passage Comprehension). Studies used a variety of instruments to measure externalizing behavior, but the most common measures were parent and teacher reports of the Child Behavior Checklist (CBCL; Achenbach, 1999) (16 effect sizes) and Strengths and Difficulties Questionnaire (SDQ; Goodman, 1997) (10 effect sizes). See Tables 1 and 2 in the Appendix B for all effect sizes separated by concurrent and longitudinal correlations, along with information about study measures.

**Table 2:** Moderators of the Effect Size Between Externalizing Behaviour and Reading

	Estimate	SE	*Z* value	*P* value	CI LB	CI UB	Significance
Continuous Moderators							
Age	-0.0228	0.0042	-5.3761	<.0001	0.2642	-0.0145	***
% Male% Poverty	0.0027	0.0007	-3.8592	0.0001	0.0013	0.004	***
% Poverty	0.0019	0.0633	0.0298	0.9763	-0.1223	0.126	NS
Time Lag	0.0152	0.0034	4.5065	<.0001	0.0086	0.0218	***
Categorical Moderators							
Rater of Behavior							
* Parent*	-0.1334	0.0251	-5.3155	<.0001	-0.1825	-0.0842	***
Teacher	-0.1992	0.0182	-10.9377	<.0001	-0.2349	-0.1635	***
Child	0.1016	0.0227	-10.9377	<.0001	-0.1461	-0.0571	***
Type of Behavior							
Externalizing	-0.1673	0.0179	-9.331	<.0001	-0.2025	-0.1322	***
Aggression	-0.1238	0.0206	-6.0092	<.0001	-0.1641	-0.0834	***
Conduct	-0.2405	0.022	-10.9105	<.0001	-0.2837	-0.1973	***
Disruptive	-0.2481	0.0292	-8.5048	<.0001	-0.3052	0.1909	***
Type of Reading	0.0179	0.016	1.1165	0.2642	-0.0135	0.0493	NS

*Note. SE *standard error, *CI LB* 95% confidence interval lower band, *CI UB* 85% confidence interval lower bound, *NS *not significant

#### Description of participants

There was a wide range in the number of participants used to calculate each correlation (*N* = 26 to *N* = 9,192), with the median number of participants equaling 286. We estimate that the total number of participants included in the meta-analysis is around 23,000 children; however, this amount cannot be exactly calculated because we do not know how much overlap in participants there is from the studies that include national samples.

The age of children included in the samples ranges between 5 years old (Kindergarten) to 12 years old (6th grade). We extracted the mean age of the samples; the average of the mean ages of the samples was toward the younger side of the range (1st -2nd grade). However, this information is difficult to interpret because the mean age does not indicate how wide the range was for each study and because we chose to handle longitudinal correlations by extracting the mean at the earlier measurement point only.

For studies that reported sex of their sample (k = 23), most recruited an evenly split male/female sample. Only two studies (Feshbach & Feshbach, 1987; Mano et al., 2017) separated correlations for males verses females and one study (Goldberg, 2004) had a completely male sample. For studies that provided information concerning the socioeconomic status of their participants (k = 19), on average, about half (47.08%) of the sample reported living in poverty. Only seven studies reported demographic information pertaining to whether their sample included English language learners. Of those seven studies that mentioned this demographic information, 3 studies reported English language learners as an exclusion criterion. The remaining four studies had a very low percentage of their sample who were learning English as a second language (i.e., less than 17%).

Though many samples included in this meta-analysis likely include participants with disabilities or who may at-risk for disabilities, few report what percentage of their sample has a disability label. Specifically, only four studies report if their participants had developmental language disorder (DLD) and all of those studies had a very small percentage of their sample that had DLD. Only one study reported correlations comparing a group with reading disability to a group without reading disability (Boetsch, 1996). Two more studies (Garwood et al., 2017; Mano et al., 2017) have very low percentages of the sample with reading disability. Only one study (Garwood et al., 2017) intentionally recruited participants with or at risk for behavior disorders. Miller et al. (2006) recruited a sample of almost half (47.3%) of participant had or were at risk for behavior disorders. The other three studies that reported behavior disorder information about their participants had samples where under 11% of the sample had a special education label. Last, only four studies reported that their sample included children with ADHD. Mano et al. (2017) and Miller et al. (2006) recruited participants that had ADHD; whereas the other two studies had less than 17% of their sample with ADHD.

### Meta-analysis results

As hypothesized, results from the meta-analysis revealed that there was a significant, negative correlation between reading and externalizing behavior, meaning that higher behavior problems scores (higher rating on measures) were associated with lower reading scores (lower performance on measures). Specifically, the overall parameter estimate of the correlation was *r *= −0.1698 (SE = 0.01, *p* < 0.0001). The prediction interval was [−0.32, −0.04], which can be interpreted to mean that there is a 95% chance that a new study not included in the meta-analysis would report a correlation between reading and externalizing behavior that fell between the listed range.

Next, we separated the data to synthesize results from the concurrent correlations (found in 28 studies) verses longitudinal correlations (found in 32 studies). For concurrent correlations, there was a significant, negative correlation between reading and behavior (*r*= −0.1845, SE = 0.01, *p* < 0.0001). Similarly for longitudinal correlations, there was a significant, negative correlation between reading and behavior (*r*= −0.1825, SE = 0.01, *p* < 0.0001). Though these correlations do not differ significantly from one another or from the overall meta-analysis that included all effect sizes, we calculated these values to answer or first research question and better describe the relation between reading and externalizing behavior measured at the same time point and with a lag between measurement periods.

To further examine the longitudinal correlations, we synthesized only the effect sizes that reported early behavior measurement to later reading (i.e., 17 effect sizes nested in 9 reports). Results indicated that the correlation was negative and significant (*r* = −0.1750, *p* < 0.0001). This indicates that for children who have poor behavior at an early age, there is evidence suggesting that their reading performance at a later age is likely to be lower than children who do not demonstrate poor behavior. Unfortunately, we only identified six effect sizes nested within four studies that reported early reading to later behavior, which meant we chose not to conduct a meta-analysis to describe the other direction of the relation. However, all correlations reported in those four studies ranged between − 0.26 and − 0.12.

### Moderator analysis results

First, we examined the heterogeneity of effect sizes using a variety of metrics to justify moderator analysis. The Cochran’s *Q* Test for Heterogeneity was significant [*Q*(87) = 590.06, *p* < 0.0001], which indicates that the effect sizes had a significant level of variation. Tau^−^squared was 0.0045 (SE = 0.001), which characterizes the magnitude and distribution of heterogeneity. Lastly, the *I*^2^ value (88.49%), which is the proportion of variance in study estimates that is due to heterogeneity, suggested there was a meaningful proportion of observed variability to explain that was not due to error. These three metrics justified the testing of moderators to explain variance in the effect sizes.

#### Measurement moderators

We coded whether the behavior rating was conducted by a parent, teacher, or child (self), and treated that as a categorical moderator. 29 reports contained this information, which allowed us to fit a model with 87 effect sizes. Results indicated that rater type was a significant moderator (*Q*_*m*_ = 154.29, *p* < 0.0001), which means the correlation between reading and externalizing behavior depended on who was the indirect rater of behavior on the measures. Teachers had the most negative correlation between reading and externalizing behavior (*r *= −0.1992), followed by parents (−0.1334) and then self-ratings by child (−0.1016). Table 2 contains results from all moderator analyses.

Next, we tested whether the type of reading (word reading, comprehension, or a combination of both) categorically moderated the effect size between reading and externalizing behavior. All 30 reports contained this information for the 88 effect sizes. This moderator was not significant (*Q*_*m*_= 1.246, *p* = 0.2642), which indicates that the type of reading assessment does not change the overall correlation between reading and externalizing behavior.

Similarly, we tested whether the type of behavior or behavior dimension (externalizing, aggression, conduct problems, disruptive behavior) categorically moderated the effect size between reading and externalizing behavior. All 30 reports contained this information for the 88 effect sizes. Results indicated that type of behavior was a significant moderator (*Q*_*m*_ =253.17, *p* < 0.0001). Disruptive behavior had the strongest negative correlation (−0.2481), closely followed by conduct problems (−0.2405). Verbal and physical aggression (−0.1238) and externalizing behavior broadly (−0.1673) had weaker but still negative and significant correlations.

For longitudinal studies only (25 effect sizes nested within 9 reports), we tested whether the time lag between measurement points was a continuous moderator of the effect size between reading and externalizing behavior. Lag between measurement points was significant and positive (*B* = 0.0152, *p* < 0.0001), meaning that longer lag time resulted in more positive (i.e., less negative) correlations.

#### Participant characteristic moderators

We extracted the mean age of the participant samples from each study and treated mean age as a continuous moderator. All 30 reports contained this information for the 88 effect sizes; however, some reports only reported an age range. In those cases, the median age of the range was coded. Notably, in longitudinal correlations, the mean age of the earliest measurement point was extracted. Results indicated that age was a significant negative moderator of effect size (*B=* −0.0228, *p* < 0.0001), indicating that as sample mean age became older, the correlation between reading and externalizing behavior became more negative.

When reported, we coded the percentage of the sample that was male (63 effect sizes in 21 reports) as a continuous moderator. Percentage male was a significant positive moderator of the correlation between reading and externalizing behavior (*B* = 0.0027, *p* < 0.0001). This means that as the sample of the studies contained more male participants, the correlation between reading and externalizing behavior became more positive (i.e., less negative).

When reported, we coded the percentage of the sample that was living in poverty (35 effect sizes in 18 reports) as a continuous moderator. This moderator was not significant (*Q*_*m*_ *=* 0.0019, *p* = 0.97), which indicates that the correlation between reading and externalizing behavior does not depend on socioeconomic status of the sample.

As part of our research questions, we wished to explore whether the association between learning English as a second language moderated the effect between reading and externalizing behavior. Unfortunately, we were unable to run this moderator because only seven studies reported this information and only three of the seven studies contained English Language Learners in their sample. Notably, the study with the highest percentage of their sample including English Language Learners (16.2%), reported correlations that ranged from − 0.19 to −0.15 (Morgan et al., 2019).

Furthermore, we wanted to explore whether relevant disability statuses of the samples moderated the correlation between reading and externalizing behavior. As reported earlier in the manuscript, very few studies (all less than four studies) included information about whether their sample had or was at risk for developing reading disability, behavior disorder, developmental language disorder, or ADHD. Thus, we were unable to examine disability as a participant characteristic as a moderator of the effect size between reading and externalizing behavior. Though we could not statistically examine the small number of studies that reported information about disability status of their sample, we have narratively summarized the findings of those particular studies. Garwood & Vernon (2017) specifically recruited participants with behavior disorders in 3rd grade. They reported a correlation of *r *= −0.17 between externalizing behavior and passage comprehension and a correlation of *r*= −0.13 for externalizing behavior and word reading. Boetsch (1996) compared a sample with reading disability to a sample without reading disability (mean age = 7.84). The correlation between reading and externalizing behavior was *r *= −0.11 for the group without reading disability and *r *= −0.07 for the group with reading disability. Mano et al. (2017) and Miller et al. (2006) both recruited samples of participants with ADHD. Twelve correlations between reading and externalizing behavior were reported in those two studies that ranged from − 0.33 to 0.02.

## Discussion

The purpose of this correlational meta-analysis was to examine the relation between externalizing behavior and reading in a diverse population. As expected, there was a negative association between externalizing behavior and reading, indicating that as students were reported as having more or higher externalizing behavior, their reading performance became lower. This association, though small (*r *= −0.1698), was significant and has several implications for future research and practice.

Prior research has synthesized effective academic interventions for students with behavior disorders (Lane, 2004; Vannest et al., 2011) and can guide educators in selecting interventions that are most appropriate for students who struggle with reading and behavior. This study emphasizes the relation between reading and behavior and the importance of considering both constructs together in a school setting. Researchers have identified effective teaching strategies, like behavior-specific praise and opportunities to respond, that can improve academic engagement for students with behavior disorders (Sutherland & Wehby, 2001). Recently, Roberts and colleagues (2023) demonstrated that evidence-based behavioral supports could increase engagement and decrease disruptive behavior when embedded into small group reading intervention. Understanding the association between reading and externalizing behavior highlights the need for educators to consider academic support when intervening on behavior and to consider behavior support when intervening on academics. Embedding behavioral supports in reading instruction has great potential for remedying deficits in both constructs and interrupting the negative relation between reading and externalizing behavior (Garwood et al., 2020).

### Concurrent verses longitudinal association

Another purpose of this meta-analysis was to examine the correlation between reading and externalizing behavior both concurrently and longitudinally. The longitudinal (or predictive) association did not differ significantly from the concurrent association. Both associations were small and negative and differed by only one-thousandth of a point (−0.1845 and − 0.1825). This indicates that there is a significant and negative correlation between reading and externalizing behavior when measured concurrently and when time has passed between measurement points. There is debate within the literature about the directionality of the relation between reading and behavior. Some studies indicate that early reading failure leads to future heightened levels of externalizing behavior (Bennet et al., 2003), likely because students cannot attend to their schoolwork and become bored and therefore act out. The cycle then repeats and worsens. Other studies suggest that early heightened externalizing behavior leads to future reading failure (Johnson et al., 2005), because students are busy misbehaving and therefore do not get adequate reading instruction. In this meta-analysis, we were able to confirm that there is a significant, negative correlation between early externalizing behavior and later reading (*r *= −0.1638). This indicates that students who exhibit externalizing behavior in early grades are at risk for poor reading performance in later elementary grades. Future studies should synthesize the correlation between early reading to later behavior. As mentioned earlier, the relation could be bidirectional (Morgan et al., 2008), but also more likely to be caused by shared cognitive factors.

### Moderators

We explored potential factors that moderated the correlation between reading and externalizing behavior because there was substantial variance among the effect sizes across studies. We sought to analyze factors related to measurement and participant characteristics to better understand for whom and under what conditions the correlation strengthens or weakens.

#### Measurement moderators

The correlation between reading and externalizing behavior depended on who scored the child’s behavior on the rating scales. Teachers reported the most negative correlation between reading and externalizing behavior, compared to how parents or children themselves rated their behavior. When rating ADHD symptomology, parents and teachers tend to have moderately correlated scores, which suggests they identify similar patterns in behavior of the children they are observing (Sullivan & Riccio, 2007). However, prior research has found that when parents and teachers rate behavioral and emotional problems in children using tools like the CBCL, their scores differ (Verhulst & Akkerhuis, 1989). Perhaps teachers have a better sense of typical child behavior performance than parents because of their exposure to many different students that can serve as comparison. It is also possible that differences between the home environment verses the school environment could explain the variation. At school, children may be asked to do more aversive tasks (e.g., reading and math) than they are asked to do at home, which could result in children engaging in externalizing behavior.

The relation between oral language and reading comprehension is well documented in the literature (e.g., Foorman et al., 2015) and is represented in the Simple View of Reading (Hoover & Gough, 1990). There has also been substantial research that documents the relation between oral language and externalizing behavior (e.g., Chow et al., 2018). Oral language ability influences how students engage and behave in the classroom (Chow & Wehby, 2019) and the correlation between oral language and externalizing behavior is significant and negative (Chow & Wehby, 2018). Because oral language is closely linked to both behavior and reading comprehension, it was expected that there would be differences between whether children were assessed on word reading or reading comprehension. Interestingly, in the current study, the type of reading did not moderate the correlation between reading and externalizing behavior. This non-significant finding could have been due to the way that reading was coded. Type of reading was sorted into three categories- word reading, comprehension, or a combination of the two constructs. Perhaps the combination category (representing almost half of the effect sizes) washed out any effects that might have been detected from examining word reading and comprehension alone.

The type or dimension of behavior that was scored moderated the association between reading and externalizing behavior, with disruptive behavior having the most negative correlation, closely followed by conduct problems. Aggression and externalizing behavior broadly still had significant, but less negative correlations with reading. It is possible that there are certain manifestations of externalizing behavior that disrupt reading instruction more than others. It is also possible that these findings are driven more by the specific tools used rather than the constructs themselves. For example, disruptive behavior was categorized only when studies used the Teacher Observation of Classroom Adaptation-Revised (TOCA-R; Werthamer-Larsson et al., 1991); furthermore, the CBCL sometimes reports aggression as a subtest and sometimes reports an externalizing behavior total score that includes aggression and rule-breaking behavior. Therefore, these results should be interpreted to mean that the relation between externalizing behavior and reading depends on how different items are grouped together to form dimensions of behavior across multiple published checklists.

When examining longitudinal correlations only, we found that longer lag between measurement periods resulted in a weaker (or not as negative) correlation between reading and externalizing behavior. It is possible that as time elapses, environmental factors have more influence. In other words, the concurrent correlation may be more precise than the longitudinal correlation because they are captured at the same time and have fewer environmental influences. Specifically for the externalizing behavior checklists completed by teachers, it is possible that the person completing the measure changes each year and in turn, distorts the correlation. Another possibility is that reading repair or behavior modification is successfully occurring due to effective intervention. For example, students who initially have poor behavior and poor reading may receive small group reading instruction that improves their reading skills, so that when they are assessed in later elementary years, the correlation between early behavior and later reading is weaker than when they were assessed concurrently or closer together.

#### Participant characteristic moderators

Several participant characteristics were treated as moderators of the correlation between reading and externalizing behavior, including mean age, percentage of the sample that was male, and percentage of the sample that come from low socioeconomic circumstances. It should be noted that these analyses are susceptible to aggregate data bias because they lack individual participant data and thus should be interpreted with caution (Geissbühler et al., 2021). Specifically, all participant characteristic moderators use sample averages and percentages of sample composition, which could in turn, lead to overestimation or underestimation of the association between participant-level data and the correlation between reading and externalizing behavior.

Mean age of the sample moderated the correlation between reading and externalizing behavior. As the mean age of the sample increased (got older), the correlation became more negative. In contrast to the theory concerning time lag as a moderator (i.e., that reading skill and behavior could be repaired over time), this evidence might indicate that as time passes, the skill deficits of students are exacerbated. This finding highlights the possible bidirectional relation between reading and externalizing behavior, with each deficit negatively worsening the other (Morgan et al., 2008). Importantly, this finding emphasizes the need for early intervention in reading (Lovett et al., 2017) and behavior (Fox et al., 2002), particularly in the lower elementary ages.

We examined sex as a continuous moderator (i.e., percentage of the sample that is male) because of limited studies that reported correlations for boys and girls separately. We found that as studies had samples that were more female, the correlation between reading and externalizing behavior became more negative. This finding was surprising because it is well documented that boys tend to exhibit more externalizing behavior than girls (e.g., Castealao, & Kröner-Herwig, 2014), particularly in young children (Boeldt et al., 2012). Furthermore, environmental factors and emotional regulation differ in explaining externalizing behavior for girls and boys (Hill et al., 2006). However, these studies do not examine that phenomenon in the context of reading skill. Two studies included in the meta-analysis examined the correlation between reading and externalizing behavior separately for girls compared to boys. Mano et al. (2017) found that in a sample of students with ADHD and reading disability, for girls only, externalizing problems were significantly related to reading. Notably, in this sample, the level of externalizing behavior was the same for boys and girls. The authors also controlled for attention, which indicates that there may be another factor that is responsible for the negative relation between reading and externalizing behavior for girls specifically. Similarly, Feshbach and Feshbach (1987) studied the relation between reading and aggression (as well as empathy and depression) and found that the relation between reading and aggression was only significant for girls, not boys. Authors from the two studies speculate that girls might be more sensitive or aware of reading deficits than boys, and in turn, become more frustrated or embarrassed, which causes them to engage in externalizing behavior. It is also possible that teacher and parent judgement of externalizing behavior is more influenced by cultural stereotypes of girls compared to boys (with boys’ hyperactivity being more socially accepted and girls’ higher academic achievement being more expected) and thus ratings of behavior may be unduly influenced (Hartley & Sutton, 2013; Horn, 2004; Whiting & Edwards, 1973).

Lastly, percentage of the sample that comes from low socioeconomic environments did not moderate the association between reading and externalizing behavior. Similarly, Chow and Wehby’s (2018) correlational meta-analysis between problem behavior and oral language reported that SES (as classified as risk status) did not moderate the correlation. Nonetheless, it is well documented that low socioeconomic status predicts reading failure (Crowe et al., 2009; Lee & Burkam, 2002) and heightened externalizing behavior (Eamon, 2000; Plybon & Kliewer, 2001), so it is unexpected that this factor had no influence on the correlation between the two constructs. Only 19 studies in the review reported information on SES, so perhaps there was not enough data or range in the data to detect this effect.

### Limitations

The current review has a handful of limitations mainly related to the studies and samples that were available to be synthesized. First, there were several participant characteristic moderators that had too few studies to examine. Specifically, we aimed to examine whether English language learner status, and inclusion of students with disabilities like reading disability, language disorder, behavior disorder, and ADHD, moderated the association between reading and externalizing behavior. It is likely that many of the samples contained students with disabilities that authors did not have information about or neglected to report, or that the students had undiagnosed issues with reading, behavior, and attention. Because there is a lack of information describing the ability level of the included samples, this meta-analysis should be viewed as describing the association between reading and externalizing behavior for a typically developing (and very heterogeneous) population. Future research should explore how this relation is strengthened or weakened when examining populations who are English language learners or have a high-incidence disability. At minimum, primary studies should strive to report demographic and ability characteristics of their samples to aid in future reviews’ examination of these variables.

Second, we were unable to control for student attention or hyperactivity in this meta-analysis. Willcutt and Pennington (2000) highlight the relation between reading and behavior and reveal attention and hyperactivity as possible underlying mechanisms for deficits in both areas. We attempted to “control” for attention and hyperactivity by examining studies that included students with ADHD diagnoses. This approach, however, does not use individual participant data on attention and hyperactivity to explain the association between reading and externalizing behavior.

Third, as with any systematic review, it is possible that we did not find all the unpublished and published articles that met our inclusion criteria. We conducted a comprehensive, multi-database search and combined results from ProQuest and PubMed; we also conducted an ancestral search. Notably, the search was run over two years ago. To alleviate this concern, we calculated a prediction interval, which describes the range for which there is a 95% chance that a newly identified study’s correlation would be reported within. Nonetheless, studies that were inadvertently excluded from the review have a chance of influencing the results of the meta-analysis.

Fourth, we imposed criteria on the type of reading (direct assessment) and behavior measures (indirect rating scales) that limit the generalizability and implications of findings beyond these measurement types. Although some studies may have measured reading using a rating scale, such as an academic performance subscale, and some studies may have conducted direct observations of behavior and correlated those measures with reading constructs, those correlations likely are connecting substantively different constructs (e.g., teacher perceptions of student reading ability versus student’s actual performance on a reading assessment). Futhermore, we did not analyze whether differences in reading assessments (i.e., standardized vs. unstandardized) moderated the relation between reading and externalizing behavior.

Last, we only conducted reliability for 33% of the studies for study coding. Though agreement was high, it is possible that the primary coder made errors while screening studies or coding study characteristics that could influence the results of the meta-analysis.

### Future directions

The results of this meta-analysis create opportunities for future research that have been discussed in paragraphs above and are more generally mentioned here. First, this meta-analysis focused specifically on elementary school students. Even with this restricted focus, the correlation between reading and externalizing behavior was strengthened as children became older. Future reviews should synthesize studies that include students in middle school and high school to determine if increasing age always strengthens the relation or if there is a level in schooling for which the correlation between reading and externalizing behavior stabilizes.

Second, this meta-analysis is restricted to studies that assessed reading in English. There are several studies conducted in other languages like Finnish (Halonen et al., 2006; Metsäpelto et al., 2017; Silinkskas et al., 2015) that reported the correlation between reading and externalizing behavior. The decision to only examine studies where participants took reading assessments in English was made because English is less orthographically transparent than other languages, specifically Finnish (Ziegler et al., 2010). Though the relation between orthography and decoding skill has been studied (Geva et al., 1993), less is known about how reading and externalizing behavior may differ across languages.

Last, the directionality of the relation between reading and externalizing behavior is still debated. This meta-analysis found that early behavior problems, in fact, are negatively associated with later reading. Future research to focus on underlying mechanisms and cognitive factors that may mediate or explain the relation. If future research can identify and examine the factors that explain the relation, then there is opportunity for interventions to disrupt the cyclical worsening of reading and externalizing behavior. This could lead to better academic and social outcomes for students as they progress through middle school and high school.

## Conclusion

In summary, we conducted a correlational meta-analysis to examine the relation between reading and externalizing behavior. We found a significant, negative correlation, which indicates that children with poor reading performance have more sympoms of externalizing behavior. This relation was strenghtend or weakened depending on rater type, behavior dimension, time between longitudinal measurement points, age of the sample, and percentage male of the sample. Understanding the relation between reading and externalizing behavior is important knowledge for both educators and researchers as they try to improve educational outcomes for students.

### Electronic supplementary material

Below is the link to the electronic supplementary material.

Supplementary Material 1 (DOCX 23.3 KB)

Supplementary Material 2 (DOCX 200 KB)
