# Supplementary material for: The relation between reading and externalizing behavior: a correlational meta-analysis
Source: Ann Dyslexia. 2024 Jun 29;74(2):158–86. doi: 10.1007/s11881-024-00307-w (PMC11249710; doi:10.1007/s11881-024-00307-w)
Supplement: Supplementary file 1 — Supplementary Material 1 (DOCX 23.3 KB) [file 11881_2024_307_MOESM1_ESM.docx]

Supplementary Information

Study Coding Manual

| **Label** | **Description** | **Response** |
| --- | --- | --- |
| Study ID | This is a number to identify the study. If the study reports two correlations from two different samples (e.g., dyslexic vs non-dyslexic) code the second correlation’s study ID with “###a.” If the study reports data from a national sample (e.g., ECLS-K) label it under the same study number. | Study ID |
| Sample | Does the sample come from a national study? If so, what is the name? | No  ECLS-K  SECCYD  Dunedin write-in |
| Authors | This is to confirm that the study ID matches the authors. | Authors |
| Year | This is to record the year the study was published | Year |
| Published | Is this study published in a peer-reviewed journal or is it a dissertation? | 1. Dissertation 2. Published |
| Correlation | Report the bivariate Pearson correlation. If the study reports correlations for subcomponents (e.g., word reading and comprehension), report two separate correlations and code them under the same study number. Do not include composite reading correlation. | Correlation |
| Number | How many participants were used to calculate this correlation? | Number |
| Longitudinal | Is this study a concurrent correlation (same time point) or a longitudinal correlation? Report all relevant correlations using the same population with the same study ID. | 1. Concurrent 2. Longitudinal |
| Direction | Code only if longitudinal. Is this correlation between early reading to later behavior or early behavior to later reading? | 1. N/A 2. Early reading to later behavior 3. Early behavior to later reading |
| Elapsed Time | Code only if longitudinal. How much time has passed between the measurement points | 1. N/A 2. Less than 1 year 3. 1 year 4. 2 years 5. 3 years 6. 4 years 7. 5 years 8. 6 years 9. 7 years |
| Reading Measure | What is the instrument used to measure reading? | 1. Woodcock Johnson 2. PIAT 3. WRAT 4. WRMT 5. GORT 6. Write in |
| Type of Reading | What construct of reading is this instrument measuring? | 1. Combination 2. Comprehension 3. Word Reading 4. Sentence completion 5. Passage fluency 6. Sentence comprehension 7. Passage Comprehension |
| Behavior Measure | What is the instrument used to measure externalizing behavior? | 1. CBCL 2. SSRS 3. BASC 4. SDQ 5. TRF 6. Write in |
| Rater | Who filled out the third-party behavior rating scale? | 1. Parent 2. Teacher 3. Child (self-assessment) |
| Type of Behavior | What construct of externalizing behavior is this instrument measuring? | 1. Externalizing 2. Aggression 3. Conduct 4. Disruptive Behavior/acting out/antisocial |
| Mean Age | What is the mean age at earliest measurement point used in the correlation? | Age   1. Kindergarten age 5-6 2. 1^st^ grade age 7 3. 2^nd^ grade age 8 4. 3^rd^ grade age 9 5. 4^th^ grade age 10 6. 5^th^ grade age 11 7. 6^th^ grade age 12 |
| **ELL** | Does the sample include any participants identified as English a second language (ESL), English Language Learner (ELL), or bilingual (as reported by the authors)? | 1. Not reported 2. No 3. Yes |
| % ELL | If yes, what % of the sample is ELL? | N/A  Not reported  Write % |
| **DLD** | Does the sample include any participants with specific language impairment or developmental language disorder or general low language skill? | 1. Not reported 2. No 3. Yes |
| % DLD | If yes, what % of the sample has DLD or low language skill? | N/A  Not reported  Write % |
| **RD** | Does the sample include any participants with specific reading disability/dyslexia? | 1. Not reported 2. No 3. Yes |
| % RD | If yes, what % of the sample has RD? | N/A  Not reported  Write % |
| **BD** | Does the sample include any participants with emotional behavior disorder or conduct disorder? | 1. Not Reported 2. No 3. Yes |
| % BD | If yes, what % of the sample has BD? | N/A  Not reported  Write % |
| **ADHD** | Does the sample include participants who are identified as having ADHD (reported by authors)? | 0-Not reported  1-No  2-Yes |
| Subtype ADHD | If yes, sample includes ADHD, what presentation is it? | 1. N/A 2. Not Reported 3. Inattention 4. Hyperactivity 5. Combined |
| % ADHD | If yes, what % of the sample has ADHD? | N/A  Not reported  Write % |
| **SES** | Does the sample claim to have participants who are from low SES (as reported by the author)? | 0-No  1-Yes |
| % SES | If yes, what % of the sample is low SES? | N/A  Not reported  Write % |
| **Sex** | Does the study report correlations for boys vs girls? | 1. No 2. All boys 3. All girls |
| **% Male** | What percentage of the sample is Male | Write percentage |
