# Supplementary material for: The relation between reading and externalizing behavior: a correlational meta-analysis
Source: Ann Dyslexia. 2024 Jun 29;74(2):158–86. doi: 10.1007/s11881-024-00307-w (PMC11249710; doi:10.1007/s11881-024-00307-w)
Supplement: Supplementary file 2 — Supplementary Material 2 (DOCX 200 KB) [file 11881_2024_307_MOESM2_ESM.docx]

**Appendix A**

**Reference List of Included Studies**

Adams, J. W., Snowling, M. J., Hennessy, S. M., & Kind, P. (1999). Problems of behaviour, reading and arithmetic: Assessments of comorbidity using the Strengths and Difficulties Questionnaire. *British Journal of Educational Psychology*, *69*(4), 571-585.

Bierman, K. L., Coie, J., Dodge, K., Greenberg, M., Lochman, J., McMohan, R., Pinderhughes, E., & Conduct Problems Prevention Research Group. (2013). School outcomes of aggressive‐disruptive children: Prediction from kindergarten risk factors and impact of the Fast Track prevention program. *Aggressive behavior*, *39*(2), 114-130.

Bodovski, K., & Youn, M. J. (2011). The long term effects of early acquired skills and behaviors on young children’s achievement in literacy and mathematics. *Journal of Early Childhood Research*, *9*(1), 4-19.

Boetsch, E. A. (1996). *A longitudinal study of the relationship between dyslexia and socioemotional functioning in young children*. University of Denver.

Boyes, M. E., Tebbutt, B., Preece, K. A., & Badcock, N. A. (2018). Relationships between reading ability and child mental health: Moderating effects of self‐esteem. *Australian Psychologist*, *53*(2), 125-133.

Braciszewski, J. E. (2007). *Intelligence, language, and behavior across the transition to reading among low-income, urban, African American youngsters* (Doctoral dissertation, Wayne State University).

Clarke, J. S. (2008). *Black children's adjustment to their parents' marital disruption: An examination of the National Longitudinal Survey of Youth (NLSY)* (Doctoral dissertation, University of Michigan).

El Nokali, N. (2012). *The intersection of physical activity, self-regulation and academic achievement: Implications for educational success* (Doctoral dissertation, University of Pittsburgh).

Feshbach, N. D., & Feshbach, S. (1987). Affective processes and academic achievement. *Child development*, 1335-1347.

Finn, J. D., Pannozzo, G. M., & Voelkl, K. E. (1995). Disruptive and inattentive-withdrawn behavior and achievement among fourth graders. *The Elementary School Journal*, *95*(5), 421-434.

Fletcher, J., Tannock, R., & Bishop, D. V. M. (2001). Utility of brief teacher rating scales to identify children with educational problems: Experience with an Australian sample. *Australian Journal of Psychology*, *53*(2), 63-71.

Garwood, J. D., Vernon-Feagans, L., & Family Life Project Key Investigators. (2017). Classroom management affects literacy development of students with emotional and behavioral disorders. *Exceptional Children*, *83*(2), 123-142.

Goldberg, S. J. (2004). *The relationship between English (L1) and Hebrew (L2) reading and externalizing behavior amongst Orthodox Jewish boys*. New York University.

Goldwater, N. (2001). *Self-cognitions and emotional and behavioural difficulties in primary school aged children with special educational needs*. University of London, University College London (United Kingdom).

Gray, S. A. (2010). *Trajectories of early externalizing behaviors: Their relation to second grade reading*. University of Massachusetts Boston.

Horan, J. M., Brown, J. L., Jones, S. M., & Aber, J. L. (2016). The influence of conduct problems and callous-unemotional traits on academic development among youth. *Journal of youth and adolescence*, *45*(6), 1245-1260.

Jeffrey, S. T. (2020). *Direct and Moderated Effects of Teacher-Child Race/Ethnic Match and Children’s Social-Emotional and Academic Development* (Doctoral dissertation, Fordham University).

Kwon, K., Kim, E., & Sheridan, S. (2012). Behavioral competence and academic functioning among early elementary children with externalizing problems. *School Psychology Review*, *41*(2), 123-140.

Magnuson, K., Duncan, G. J., Lee, K. T., & Metzger, M. W. (2016). Early school adjustment and educational attainment. *American educational research journal*, *53*(4), 1198-1228

Mano, Q. R., Jastrowski Mano, K. E., Denton, C. A., Epstein, J. N., & Tamm, L. (2017). Gender moderates association between emotional-behavioral problems and text comprehension in children with both reading difficulties and ADHD. *Psychology in the Schools*, *54*(5), 504-518.

Martoccio, T. L. (2014). *Multilevel Latent Class Analysis for the identification of preschool-aged children's internal representation typologies and early parenting predictors in a low-income sample*. Michigan State University.

Mesite, L. (2019). *Exploring Gender Differences in Children's Early Reading Development in the US* (Doctoral dissertation, Harvard University).

Miller, C. J., Miller, S. R., Trampush, J., McKay, K. E., Newcorn, J. H., & Halperin, J. M. (2006). Family and cognitive factors: Modeling risk for aggression in children with ADHD. *Journal of the American Academy of Child & Adolescent Psychiatry*, *45*(3), 355-363.

Morrison, D., Mantzicopoulos, P., & Carte, E. (1989). Preacademic screening for learning and behavior problems. *Journal of the American Academy of Child & Adolescent Psychiatry*, *28*(1), 101-106.

NICHD Early Child Care Research Network. (2005). Early child care and children’s development in the primary grades: Follow-up results from the NICHD Study of Early Child Care. *American Educational Research Journal*, *42*(3), 537-570.

Razza, R. A., Martin, A., & Brooks-Gunn, J. (2015, December). Are approaches to learning in kindergarten associated with academic and social competence similarly?. In *Child & youth care forum* (Vol. 44, No. 6, pp. 757-776). Springer US.

Sasser, T. R., Bierman, K. L., & Heinrichs, B. (2015). Executive functioning and school adjustment: The mediational role of pre-kindergarten learning-related behaviors. *Early childhood research quarterly*, *30*, 70-79.

Stormont, M., Cohen, D. R., Herman, K. C., & Reinke, W. M. (2019). Teacher-rated school readiness items in a kindergarten sample: Outcomes in first grade. *School Psychology*, *34*(6), 612.

Swanson, J., Valiente, C., Bradley, R. H., Lemery‐Chalfant, K., & Abry, T. (2016). Teachers’ effortful control and student functioning: Mediating and moderating processes. *Social Development*, *25*(3), 623-645.

Tang, X., & Dai, T. (2021). How do classroom behaviors predict longitudinal reading achievement? A conditional autoregressive latent growth analysis. *Early Childhood Research Quarterly*, *54*, 239-251.

Tomblin, J. B., Zhang, X., Buckwalter, P., & Catts, H. (2000). The association of reading disability, behavioral disorders, and language impairment among second‐grade children. *Journal of child Psychology and Psychiatry*, *41*(4), 473-482.

Wang, X. (2009). *Maternal education, maternal language acculturation, parental involvement, and maternal social support as predictors of the academic achievement and socioemotional development of Asian American children*. University of Maryland, College Park.

**Appendix B**

Table 1

*Concurrent Effect Sizes*

*Note.* ECLS-K= Early Childhood Longitudinal Studies Kindergarten, NSYL= National Longitudinal Survey of Youth, CBCL= Child Behavior Checklist, ACBC= Achenbach Child Behavior Checklist, TRF= Teacher's Report Form, SSRS= Social Skills Rating System, TOCA= Teacher Observation of Classroom Adaptation, SDQ= Strengths and Difficulties Questionnaire, CAS= Children's Aggression Scale, MacArthur HBQ= MacArthur Health and Behavior Questionnaire, Aggression Scale (Orpinas & Frankowski, 2001), BASC- Behavior Assessment System for Children, BPC= Behavior Problem Checklist, BDHI= Buss-Durkee Hostility Inventory, BPI= Behavior Problems Inventory-CRS= Teacher-Child Rating Scale Combination= both word reading and comprehension

Table 2

*Longitudinal Effect Sizes*

*Note.* ECLS-K= Early Childhood Longitudinal Studies Kindergarten, CBCL= Child Behavior Checklist, SPQ= Student Participant Questionnaire, TRF= Teacher's Report Form, SSRS= Social Skills Rating System, TOCA= Teacher Observation of Classroom Adaptation, MacArthur HBQ= MacArthur Health and Behavior Questionnaire, Aggression Scale (Orpinas & Frankowski, 2001), BASC- Behavior Assessment System for Children, Combination= both word reading and comprehension
